# Impact of intrauterine infusion of human umbilical cord blood mononuclear cells on reproductive outcomes in patients with thin endometrium undergoing frozen embryo transfer

**DOI:** 10.3389/fcell.2026.1758224

**Published:** 2026-05-28

**Authors:** Rui Xing, Gui-Min Hao, Yan-Jing Wu, Xiao-Qi Zuo, Pan Zhao, Chun-Hui Mi, Cong-Pin Yang, Kun Jia, Ai-Min Yang

**Affiliations:** 1 Department of Reproductive Medicine, The Second Hospital of Hebei Medical University, Shijiazhuang, China; 2 Hebei Center for Quality Control and Management of Human Assisted Reproductive Technology, Shijiazhuang, China; 3 Hebei Key Laboratory of Infertility and Heredity, Shijiazhuang, China; 4 Department of Ultrasound, Hebei General Hospital, Shijiazhuang, China

**Keywords:** frozen embryo transfer, human umbilical cord blood mononuclear cells, intrauterine adhesion, live birth, thin endometrium

## Abstract

**Objective:**

This study aims to assess the effects of intrauterine infusion of human umbilical cord blood mononuclear cells (HUCBMCs) on reproductive outcomes in patients with thin endometrium undergoing frozen embryo transfer (FET).

**Design:**

Prospective cohort study.

**Study Participants:**

This study enrolled 134 patients diagnosed with thin endometrium who underwent FET procedures between January 2022 and December 2023. The cohort was stratified into two groups: a treatment group receiving HUCBMCs infusion (n = 35) and a control group (n = 99).

**Methods:**

Participants were allocated into two groups: the HUCBMCs group and the control group, based on the administration of HUCBMCs intrauterine infusion. Reproductive outcomes were monitored for all participants. The primary outcome measure was live birth, while secondary outcomes encompassed endometrial characteristics (thickness, pattern, and endometrial–subendometrial (ES) blood flow distribution pattern), implantation rate, biochemical pregnancy, clinical pregnancy, ongoing pregnancy, miscarriage rate, and obstetric outcomes. Multivariable logistic regression analyses were employed to ascertain the independent effects of HUCBMCs.

**Results:**

No significant improvement in endometrial characteristics was noted following the infusion of HUCBMCs. Similarly, the live birth rate did not differ significantly between the groups, with the HUCBMCs group exhibiting a live birth rate of 27.6% (8 out of 29 patients), comparable to the control group 27.1% (19 out of 70 patients) (*P* = 0.964). Additionally, when compared to the control group, the HUCBMCs group showed no statistically significant differences in implantation rate, biochemical pregnancy, clinical pregnancy, and ongoing pregnancy, as determined by multivariate logistic regression analysis (OR 0.46, 95% CI 0.16–1.32; OR 0.74, 95% CI 0.24–2.26; OR 0.79, 95% CI 0.26–2.41; OR 0.96, 95% CI 0.13–7.22, respectively). Notably, the miscarriage rates were 38.5% in the HUCBMCs group and 36.7% in the control group. Moreover, blastocyst transfer was associated with a significantly higher likelihood of live birth compared to cleavage embryo transfer, as evidenced by both crude and multivariate logistic regression analyses (OR 6.52, 95% CI 2.22–19.20; OR 7.86, 95% CI 2.20–28.08). No adverse effects were observed in the HUCBMCs group.

**Conclusion:**

Our findings indicate that although HUCBMCs infusion was deemed safe, there was no statistically significant improvement in reproductive outcomes between the HUCBMCs group and the control group. The miscarriage rate remained high among patients with thin endometrium. However, blastocyst transfer may be associated with a higher live birth rate compared to cleavage embryo transfer.

## Introduction

Successful embryo implantation necessitates both high-quality embryos and a receptive endometrium. A thin or compromised endometrium poses a substantial obstacle to achieving a successful pregnancy. Thin endometrium is characterized by an endometrial thickness of less than 7 mm as outlined in the guidelines from the Canadian Fertility and Andrology Society and the Chinese expert consensus from the Society of Reproductive Medicine, Chinese Medical Association (Du et al., 2020). An endometrial thickness (EMT) of less than 7 mm is generally deemed suboptimal for embryo implantation (Craciunas et al., 2019). Several factors, such as repeated uterine procedures, infections, medication effects, and primary causes, contribute to the development of a thin endometrium. The development of intrauterine adhesion following uterine cavity procedures is the primary cause of a thin endometrium, which is defined as the formation of fibrotic scar tissue within the uterine cavity.

Conventional treatments for thin endometrium encompass the administration of exogenous estrogen, interventions aimed at enhancing uterine blood flow (such as low-dose aspirin, sildenafil citrate, and low-molecular-weight heparin), and platelet-rich plasma (PRP) therapy (Shin et al., 2024; Zhang et al., 2022; Li et al., 2020; Vaidakis et al., 2024). Nonetheless, patients with thin endometrium exhibit inadequate responses to these conventional treatments. HUCBMCs represent a heterogeneous assembly of immune cells and pluripotent stem cells. This population predominantly consists of hematopoietic stem cells, mesenchymal stem cells, and neural stem cells, in addition to various immune cell types such as natural killer cells, T lymphocytes, dendritic cells, and regulatory T cells. Together, these diverse cellular components contribute to the complex and varied cell populations characteristic of HUCBMCs. HUCBMCs serve as a crucial source of hematopoietic stem cells and are extensively utilized in regenerative medicine for tissue and organ repair (Bae et al., 2025; Li et al., 2024). Moreover, previous investigation found that HUCBMCs had potential role of induction of angiogenesis (Gatina et al., 2023). In rat models, HUCBMCs injections had the ability to differentiate into epithelial, vascular endothelial, and ER cells; enhance blood supply; inhibit fibrosis; and restore fertility in the IUA model (Zheng et al., 2020). Therefore, the application of HUCBMCs is thought to be a promising approach in treating thin endometrium. Nonetheless, the therapeutic efficacy of HUCBMCs in addressing thin endometrium has not been definitively established. This study seeks to assess the clinical efficacy of HUCBMCs in patients with thin endometrium undergoing FET.

## Materials and methods

### Study participants and design

This prospective cohort study was approved by the Ethics Committee of the Second Hospital of Hebei Medical University (2022-T005). A total of 134 infertile patients with thin endometrium who underwent frozen embryo transfer (FET) from January 2022 to December 2023 were included. All participants provided written informed consent for the use of their medical records prior to treatment. Participants were divided into two groups: the HUCBMCs group and the control group, based on whether they received HUCBMCs infusion.

The inclusion criteria were as follows: 1) patients aged between 20 and 40 years; 2) all patients met the diagnostic criteria for thin endometrium, defined as an endometrial thickness of less than 7 mm on the day of ovulation, the day of human chorionic gonadotropin (HCG) injection in fresh *in vitro* fertilization (IVF) cycles, or the day of progesterone initiation in frozen-thaw embryo transfer cycles; and 3) availability of at least one frozen embryo for transfer, including both cleavage-stage embryos and blastocysts.

The exclusion criteria included uterine malformations (such as unicornuate, septate, or bicornuate uterus), untreated endometrial lesions (including polyps, hyperplasia, or intrauterine adhesions), uterine fibroids distorting the uterine cavity diagnosed via transvaginal ultrasound (TVS) or hysteroscopy, adenomyosis, chromosomal abnormalities in either male or female partner, patients undergoing a donor oocyte program or preimplantation genetic testing (PGT), recurrent spontaneous abortion (RSA), untreated tubal hydrosalpinx and allergies or contraindications to HUCBMCs.

### Ultrasound investigation

Endometrial thickness was measured using a colour Doppler ultrasound system (GE Voluson E10) via transvaginal ultrasound. All ultrasound scans were performed by two experienced doctors. The hormone replacement therapy (HRT) protocol and gonadotropin-Releasing Hormone analogue (GnRHa) pretreatment combined with HRT were assessmented on the day of progesterone supplementation, while the natural cycle (NC) protocol and the ovulation induction (OI) protocol were assessmented on the day of ovulation. Once a satisfactory longitudinal view of the uterus was achieved, the colour Doppler mode was activated. The area of interest was the endometrium and the subendometrial regions within 10 mm of the echogenic endometrial borders. Identical colour Doppler settings were maintained constant for all participants to ensure standardization of the examinations.

**FIGURE 1:**
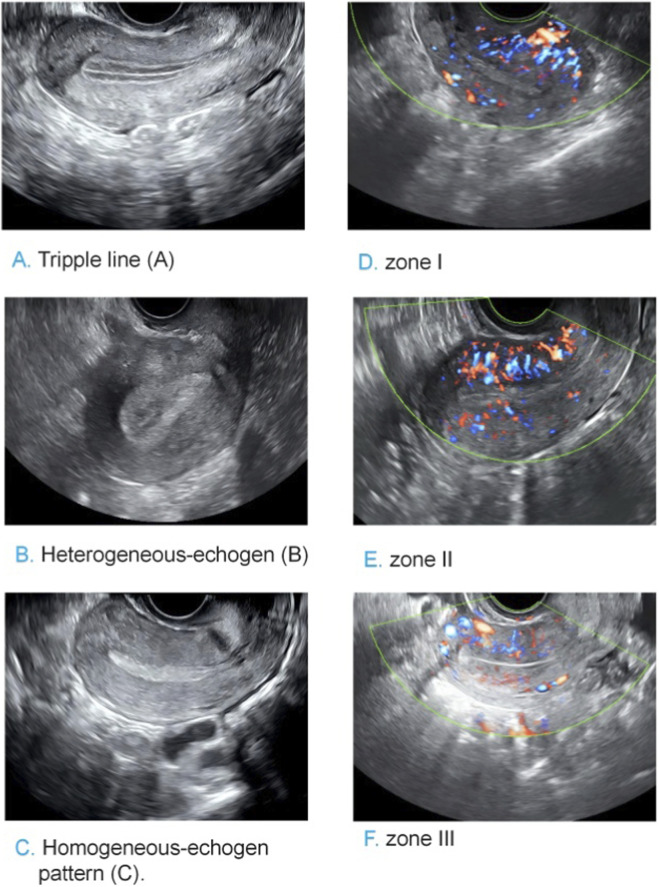
The endometrium pattern and endometrial–subendometrial blood flow distribution pattern Endometrial pattern was classified as: **(A)** pattern A: triple line, **(B)** pattern B heterogeneous-echogen and **(C)** pattern C homogeneous-echogen. **(D)** Zone I, vessels penetrating the outer hypoechogenic area surrounding the endometrium but not entering the hyperechogenic outer margin, **(E)** zone II, vessels penetrating the hyperechogenic outer margin of the endometrium but not entering the hypoechogenic inner area, **(F)** zone III, vessels entering the hypoechogenic inner area.

We adopted the definition from Applebaum (1995), summarized as follows: zone I, vessels penetrating the outer hypoechogenic area surrounding the endometrium but not entering the hyperechogenic outer margin; zone II, vessels penetrating the hyperechogenic outer margin of the endometrium but not entering the hypoechogenic inner area; and zone III, vessels entering the hypoechogenic inner area. Endometrial pattern was classified as three types: pattern A, triple line; pattern B, heterogeneous-echogen; pattern C, homogeneous-echogen (Ahmadi et al., 2017) (Figure 1).

### The isolation of HUCBMCs

HUCBMCs are commercial products of Shandong Qilu Stem Cell Engineering Co., Ltd. In accordance with the provided guidelines, the preparation procedure is delineated as follows:

Umbilical cord blood from healthy donors, who provided informed consent post-delivery, was utilized in this study. The donor inclusion criteria are as follows: 1) Mother’s age ≥ 18 years old; 2) Single pregnancy; 3) Voluntarily participate and sign the informed consent form. While the donor exclusion criteria included (any one of the following): 1) There is a family history of genetic diseases; 2) The mother has a history of infectious diseases, including but not limited to: HIV-1/2, HTLV-1/2, hepatitis B (HBsAg positive), hepatitis C (HCV antibody positive), and syphilis (serological test positive); 3) The mother had active infections such as cytomegalovirus (CMV) and rubella virus during her pregnancy; 4) The mother has a history of malignant tumor; 5) The mother has a history of autoimmune diseases; 6) The mother has a long history of drug abuse; 7) There were significant congenital malformations or chromosomal abnormalities at birth; 8) Gestational age less than 37 weeks (premature birth).

All donors underwent detailed health questionnaires and serological screening for the above-mentioned infectious diseases both before and after delivery. Only the umbilical cord blood of donors who passed all the screenings will be used for the preparation of HUCBMC. Follow-up health assessments were conducted at 1-, 2-, and 3-year post-collection to monitor donor health and maintain traceability of the cell sources.

Umbilical cord blood was collected from the umbilical vein immediately after delivery into sterile blood bags containing sodium heparin as an anticoagulant, following strict aseptic techniques. All samples were processed within 6 h of collection. Mononuclear cells were isolated by density gradient centrifugation using Ficoll-Paque (GE Healthcare, USA). Briefly, UCB was diluted with sterile normal saline, carefully layered over Ficoll-Paque, and centrifuged at room temperature. The mononuclear cell layer (buffy coat) was collected, washed twice with sterile normal saline, and resuspended in saline for further analysis.

Cell counting and viability assessment were performed using trypan blue exclusion. Cell phenotype was characterized by flow cytometry using antibodies against CD34, CD45, CD73, CD90, and CD105. Quality control tests included sterility (aerobic and anaerobic cultures), endotoxin testing (limulus amebocyte lysate assay), and colony-forming unit (CFU) assays. Cells were then resuspended in a cryoprotective solution consisting of commercial cell freezing medium supplemented with autologous umbilical cord plasma, aliquoted into sterile cryovials, and cryopreserved in liquid nitrogen (−196 °C) until use. For intrauterine infusion, cryopreserved HUCBMCs were thawed rapidly in a 37 °C water bath, washed to remove cryoprotectant, and resuspended in sterile saline at a concentration of 1 × 10^7^ cells/mL. Cell viability post-thaw was confirmed to be >90% prior to clinical use (More details were shown in Supplementary Table S2).

HUCBMCs are commercially available products manufactured by Shandong Qilu Stem Cell Engineering Co., Ltd. The accompanying product manual and quality inspection report delineate essential specifications, encompassing sterility verification, phenotypic characterization, and handling protocols. Sterility assessments for HUCBMCs encompass the following evaluations: pathogen detection (including HBsAg, HCV, syphilis, HIV I/II, and CMV-IgM); microbial culture testing (for both aerobic and anaerobic organisms); *mycoplasma* detection; and bacterial endotoxin testing. Additional phenotypic markers of HUCBMCs including activity detection (activity > 92.9%) and CFU-GEMM (colony growth is present), beyond CD34. The isolated HUCBMCs were quantified to have a concentration of 5.87 × 10^7^ cells per milliliter and a CD34+cell population of 0.36%.

### Recovery and washing process of HUCBMCs

To elucidate the procedural methodologies, we have detailed the pre-thawing and cleansing protocol for HUCBMCs in accordance with the product manual.

1. Pre-equilibrate the constant temperature water bath to 37 °C.
2. Perform ultraviolet sterilization of the laminar flow hood prior to use. Within the sterilized environment, prepare clinically required infusion vials (containing normal saline) and maintain them in a ready state.
3. Retrieve the cryopreservation tube containing cells from liquid nitrogen storage. Immediately immerse the tube in the 37 °C water bath and subject it to continuous agitation to accelerate thawing of the cell suspension. Remove the tube upon complete thawing.
4. Following complete thawing, disinfect the tube opening and external surface using a 75% ethanol-saturated cotton ball. Subsequently, transfer the disinfected tube into the laminar flow hood.
5. Under aseptic conditions, remove the tube cap. Using a sterile syringe, aspirate the cell suspension from the cryopreservation tube and promptly inject it into the prepared, disinfected infusion vial (0.5% albumin 2 mL).
6. Gently agitate the container to ensure homogeneous cell distribution. It was centrifuged at 1,200 rpm for 10 min, and the precipitate was taken. Then, the precipitate solution was mixed with 0.5% albumin. 0.5% albumin consist of albumin and saline.
7. Transfer the revived cells to the responsible nurse for clinical administration.

### Intrauterine infusion of HUCBMCs

In the HUCBMCs group, endometrial scratching was performed on menstrual cycle days 3 ∼ 4 using a disposable endometrial sampler (Pipelle de Cornier, 3.6 mm outer diameter). Immediately following the scratching procedure, 2 mL of HUCBMCs was infused via an intrauterine insemination catheter (Cook K-JETS-7019-SIV). Patients were instructed to maintain a position of pelvic elevation for 30 min post-infusion to facilitate cellular adhesion. Patients in the control group, only endometrial scratching was performed.

The patient was monitored in the hospital for 2 h following an intrauterine infusion to observe vital signs. All complaints of discomfort, including uterine perforation and anaphylactic rash, were documented in this study. The patient’s body temperature, hemogram (including leukocyte count and serum C-reactive protein levels), and vaginal discharge were recorded oneweek post-treatment.

Postoperative body temperatures, hematological parameters, and vaginal discharge were within normal limits. The mean white blood cell count (6.6 ± 1.3 × 109/L), neutrophil percentage (62.3% ± 6.3%), and C-reactive protein level (2.3 ± 0.4 mg/L) at 1 week post-treatment were observed to be within the normal range (Supplementary Table S2).

### FET treatment procedures

Four endometrial preparation protocols were employed for FET treatment: the natural cycle (NC) protocol, hormone replacement therapy (HRT) protocol, Gonadotropin-Releasing Hormone analogue (GnRHa) pretreatment combined with HRT, and the ovulation induction (OI) protocol. The NC protocol was employed for women exhibiting spontaneous ovulation, with embryo transfer occurring 3 days post-ovulation or 4 days following the LH surge for cleavage-stage embryos, and 5 days post-ovulation or 6 days following the LH surge for blastocysts. The HRT or OI protocols were administered to women experiencing amenorrhea, irregular menstrual cycles, or normal ovulation. In instances of endometriosis, the GnRHa + HRT protocol was implemented. For the HRT cycle, oral estradiol valerate (Progynova, Bayer, Leverkusen, Germany) was initiated on Days 3–4 at a dosage of 4 mg daily, subsequently increased to 6 mg daily in the following days. Depending on the thickness of the endometrium, vaginal estradiol (Femoston, Abbott, Veerweg, the Netherlands) at a dosage of 1 ∼ 2 mg daily might also be introduced. The duration of endometrial preparation spanned 12 ∼ 18 days.

Luteal support was provided to all patients following embryo transfer. In the HRT and GnRHa + HRT protocols, patients received 10 mg of dydrogesterone (Daphton, Solvay Pharmaceuticals, Netherlands) twice daily, in conjunction with 90 mg of Crinone vaginal progesterone gel (Crinone, Fleet Laboratories Limited, United Kingdom) once daily. In the NC and OI protocols, 20 mg of dydrogesterone was administered daily.

### Study variables and outcome measures

The study examined various variables and outcome measures, with baseline demographic data collected for each patient, including age, body mass index (BMI), type and duration of infertility, No (number) of previous failed embryo transfer (ET) cycles, No. of previous curettages, and causes of thin endometrium. Clinical variables assessed encompassed endometrial preparation protocols, endometrial thickness (EMT), endometrial pattern, and endometrial and subendometrial (ES) blood flow distribution pattern on the day of progesterone administration (or ovulation day), as well as the stage and number of embryos transferred, and the proportion of patients with good quality embryo transfers in both groups. For cleavage embryos, Grade 1 and Grade 2 embryos were classified as good-quality embryos (Aldemir et al., 2020); for blastocysts, a grade of ≥ 3BB (including AA, AB, BA, and BB) was defined as good-quality embryos (Gardner et al., 2000). Serum human chorionic gonadotropin (hCG) levels were measured on Day 14, and transvaginal ultrasound (TVS) was conducted 4 weeks post-embryo transfer.

The implantation rate was determined by dividing the number of gestational sacs observed on ultrasound by the number of embryos transferred. Biochemical pregnancy was defined as a serum hCG level ≥5.3 mIU/mL 14 days post-embryo transfer. Clinical pregnancy was confirmed by the observation of an intrauterine gestational sac on transvaginal ultrasound or villous tissue confirmed by histology. Patients were monitored until the end of their pregnancy by a dedicated team at our center. The complications during pregnancy, the delivery mode, gestational age (GA) at delivery, fetal survival, and neonatal birth weight were collected via phone or fax follow-up. Early miscarriage was defined as spontaneous abortion before 12 weeks. Ongoing pregnancy was defined as a viable intrauterine pregnancy of at least 12 weeks’ duration confirmed by ultrasound. Late miscarriage was defined as spontaneous abortion between 12 and 28 weeks of gestation. Preterm birth was defined as delivery between 28 and 37 weeks of gestation. Low birth weight (LBW) was defined as a birth weight of <2,500 g. Live birth was defined as the birth of at least one living child.

The primary outcome was the live birth (defined as at least one live birth after 28 weeks of gestation). Secondary outcomes included endometrial characteristics (thickness, pattern, ES blood flow distribution pattern), implantation rate, biochemical pregnancy, clinical pregnancy, ongoing pregnancy, miscarriage rate and obstetric outcomes.

### Statistical analysis

No formal *a priori* sample size calculation was performed, as this was an exploratory study aimed at generating preliminary data on the safety and potential efficacy of HUCBMCs infusion in patients with thin endometrium. Post hoc power calculations were conducted using G*Power 3.1 to contextualize the findings, based on the observed event rates in this cohort.

Demographic characteristics, clinical variables, and reproductive outcomes were compared between the two groups. Statistical analyses were performed using SPSS version 26.0 (SPSS, Inc., Chicago, IL, USA) and R language. The normality of continuous variables was assessed using the Kolmogorov-Smirnov test and Q-Q plots. Normally distributed data were presented as means with standard deviations (SDs), while non-normally distributed data were expressed as medians with interquartile ranges (25th percentile–75th percentile).

For comparisons of normally distributed data, independent samples t-test was used. The Mann-Whitney U test was applied for non-normally distributed continuous variables. Categorical data were presented as numbers with percentages, and comparisons between categorical variables were performed using the Chi-square test or Fisher’s exact test, as appropriate.

Logistic regression analysis was applied to determine the effects of each variable on live birth. Crude and adjusted logistic regression analysis were used to determine the independent effect of HUCBMCs on reproductive outcomes (implantation rate, biochemical pregnancy, clinical pregnancy, ongoing pregnancy, miscarriage rate and live birth). Variable selection was performed using the Akaike Information Criterion (AIC) to identify the most parsimonious model while maintaining predictive accuracy. The stepwise selection algorithm was employed with bidirectional elimination (both forward and backward selection) based on AIC values.

The criterion for variable retention was set at a reduction in AIC value of ≥2 points, which indicates substantially improved model fit. Variables that did not contribute significantly to model performance were sequentially removed to prevent overfitting and enhance model interpretability. These five variables were age, BMI, etiology of thin endometrium, endometrial pattern and the type of embryo transferred. In addition, before conducting multivariable regression analyses, we used univariate regression analysis to assess the independent effect of each confounder on live birth. Previous failed embryo transfer cycles did not significantly affect live birth (OR 0.85, 95% CI 0.55–1.30, P = 0.446) (Supplementary Table S1). Furthermore, considering the restricted sample size within our investigation, the incorporation of superfluous covariates has the potential to diminish the statistical efficacy of the model. Consequently, the failed embryo transfer cycles were omitted from the multivariable regression analysis. Odds ratios (ORs) and their 95% confidence intervals (CIs) were calculated to assess the strength of associations. A *P*-value of <0.05 was considered statistically significant.

## Results

### Study Participants

**FIGURE 2:**
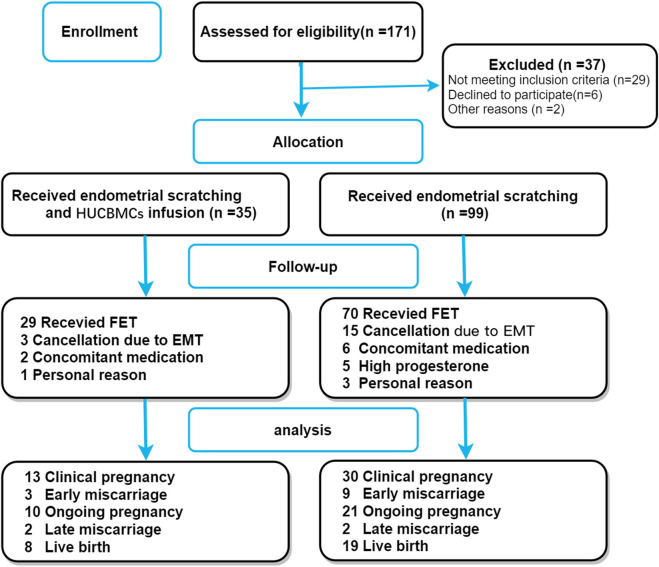
Details of flowchart of participants and main reproductive outcomes of the enrolled patients EMT: endometrial thickness; FET: frozen embryo transfer; HUCBMCs: Human Umbilical Cord Blood Mononuclear Cells.

A total of 171 patients were assessed for eligibility. 35 patients received endometrial scratching and HUCBMCs infusion, while 99 patients received endometrial scratching alone and served as the control group. 6 patients in the HUCBMCs group and 29 patients in the control group canceled embryo transfer. In the HUCBMCs group, 3 cases were canceled due to thin endometrium, 2 cases due to concomitant medication, and 1 due to personal reasons. In the control group, 15 cases were canceled due to thin endometrium, 6 due to concomitant medication, 3 due to personal reasons, and 5 due to high progesterone. 29 patients in the HUCBMCs group and 70 patients in the control group underwent embryo transfer. The flow of patient inclusion is depicted in Figure 2.

Baseline characteristics of the participants between the two groups were summarized in Table 1. No significant difference was observed between the two groups in terms of age [32.0 (28.5, 36.5) *versus* 33.0 (30.0, 36.3)], BMI [23.6 (3.2) *versus* 23.7 (3.3)], infertility type, duration of infertility [2.0 (1.0, 3.5) *versus* 2.0 (1.0, 5.0)], No. of previous curettages [1.0 (0, 2.0) *versus* 1.0 (0, 3.0)], or the etiology of thin endometrium (Table 1). The etiology of thin endometrium was classified into two categories: Traumatic factors consisted of mechanical endometrial trauma, such as curettage, myomectomy, or septotomy, while the other causes included a history of uterine artery embolization, endometrial tuberculosis, uterine anomalies, or unknown causes. The etiology of thin endometrium between the two groups demonstrated no difference. Over half of the patients in both groups had a history of iatrogenic trauma (72.4% in the HUCBMCs group *versus* 64.3% in the control group). The number of failed ET cycles in the HUCBMCs group was higher than the control group [1.0 (0, 3.0) *versus* 0 (0, 1.0)].

**TABLE 1:** Baseline characteristics of the participants in the two groups.

Characteristics	HUCBMCs (n = 29)	Control (n = 70)	*P*
Age (years)	32.0 (28.5.36.5)	33.0 (30.0.36.3)	0.265
BMI (kg/㎡)	23.6 (3.2)	23.7 (3.3)	0.914
Infertility type			0.813
Primary, n (%)	6.0 (20.7%)	16.0 (22.9%)	​
Secondary, n (%)	23.0 (79.3%)	54.0 (77.1%)	​
Infertility duration (years)	2.0 (1.0, 3.5)	2.0 (1.0, 5.0)	0.363
No. of previous curettages	1.0 (0, 2.0)	1.0 (0, 3.0)	0.270
Etiology of thin endometrium			0.435
Traumatic, n (%)	21 (72.4%)	45 (64.3%)	​
Other causes, n (%)	8 (27.6%)	25 (35.7%)	​
Failed ET cycles	1.0 (0, 3.0)	0.0 (0, 1.0)	<0.01

Categorical variables were presented as n (%) and calculated by Pearson’s chi square test or Fisher’s exact test as appropriate. Continuous data was presented as mean ± SD (normally distributed) and median (25th percentile-75th percentile) (non-normally distributed). Independent samples t-test or Mann-Whitney U Test was used for the comparisons of continuous variables between groups.

HUCBMCs: Human Umbilical Cord Blood Mononuclear Cells; BMI, body mass index; NO., number; ET, embryo transfer.

Other causes of thin endometrium include uterine artery embolization, endometrial tuberculosis, uterine idiopathic.

### Endometrial characteristics pre-HUCBMCs and post HUCBMCs infusion

After HUCBMCs intrauterine infusion, the endometrial characteristics, including EMT, endometrial pattern and ES blood flow distribution pattern, had no significant improvement (Table 2).

**TABLE 2:** Endometrial characteristics Pre-HUCBMCs and Post-HUCBMCs infusion.

Endometrial characteristics	Pre-HUCBMCs (n = 29)	Post-HUCBMCs (n = 29)	*P*
EMT (mm)	6.7 (6.0, 7.0)	6.9 (6.2, 7.4)	0.198
Endometrial pattern	​	​	0.090
Pattern A	9 (31.0%)	11 (37.9%)	​
Pattern B	13 (44.8%)	17 (58.6%)	​
Pattern C	7 (24.1%)	1 (3.4%)	​
ES blood flow distribution pattern	​	​	0.642
Zone I	7 (24.1%)	9 (31.0%)	​
Zone II	13 (44.8%)	14 (48.3%)	​
Zone III	9 (31.0%)	6 (20.7%)	​

Categorical variables were presented as n (%) and calculated by Pearson’s chi square test or Fisher’s exact test as appropriate. Non-normally distributed data was presented as median (25th percentile-75th percentile). Mann-Whitney U Test was used for the comparisons of continuous variables between groups.

HUCBMCs: Human Umbilical Cord Blood Mononuclear Cells; EMT, endometrial thickness; ES, endometrial–subendometrial.

Endometrial pattern was classified as three types: pattern A, triple line; pattern B, heterogeneous-echogen; pattern C, homogeneous-echogen.

Zone I, vessels penetrating the outer hypoechogenic area surrounding the endometrium but not entering the hyperechogenic outer margin; zone II, vessels penetrating the hyperechogenic outer margin of the endometrium but not entering the hypoechogenic inner area; and zone III, vessels entering the hypoechogenic inner area.

### Pregnancy and obstetric outcomes in HUCBMCs and control groups

No significant difference was observed in EMT between the two groups [6.9 (6.2, 7.4) *versus* 7.0 (6.5, 7.1), p = 0.819]. Furthermore, endometrial characteristics, including endometrial pattern and ES blood flow distribution pattern, did not differ significantly between the groups (refer to Table 3). Additionally, the FET protocols were comparable between the groups. However, in the HUCBMCs group, the blastocyst transfer rate was significantly higher than in the control group (69.0% *versus* 44.3%, p = 0.025). There were no significant differences in the number of embryos transferred [1.0 (1.0, 2.0) *versus* 1.0 (1.0, 2.0), p = 0.937] or in the rate of good quality embryos (55.2% *versus* 60.0%, p = 0.675) (Table 3).

**TABLE 3:** Clinical variables and pregnancy outcomes in the HUCBMCs group and the control group.

Pregnancy outcomes	HUCBMCs (n = 29)	Control (n = 70)	*P*
EMT (mm)	6.9 (6.2, 7.4)	7.0 (6.5, 7.1)	0.819
Endometrial pattern	​	​	0.065
Pattern A	11 (37.9%)	43 (61.4%)	​
Pattern B	17 (58.6%)	24 (34.3%)	​
Pattern C	1 (3.4%)	3 (4.3%)	​
ES blood flow distribution pattern	​	​	0.128
Zone I	9 (31.0%)	10 (14.3%)	​
Zone II	14 (48.3%)	37 (52.9%)	​
Zone III	6 (20.7%)	23 (32.9%)	​
FET protocols	​	​	0.258
HRT	25 (86.2%)	49 (70%)	​
GnRHa + HRT	1 (3.4%)	6 (8.6%)	​
OI	3 (10.3%)	8 (11.4%)	​
NC	0 (0%)	7 (10.0%)	​
Patients with good quality embryos, n (%)	16 (55.2%)	42 (60.0%)	0.675
No. of embryos transferred	1.0 (1.0.2.0)	1.0 (1.0.2.0)	0.937
Stage of embryos transferred	​	​	0.025
Cleavage embryo, n (%)	9 (31.0%)	39 (55.7%)	​
Blastocyst, n (%)	20 (69.0%)	31 (44.3%)	​
Reproductive outcomes			
Implantation rate, % (n)	31.0% (13/42)	29.4% (30/102)	0.854
Biochemical pregnancy rate, % (n)	44.8% (13/29)	44.3% (31/70)	0.961
Clinical pregnancy rate, % (n)	44.8% (13/29)	42.9% (30/70)	0.857
Miscarriage rate, % (n)	38.5% (5/13)	36.7% (11/30)	1.000
Early miscarriage rate, % (n)	23.1% (3/13)	30.0% (9/30)	0.727
Late miscarriage rate, % (n)	15.4% (2/13)	6.7% (2/30)	0.572
Ongoing pregnancy rate, % (n)	76.9% (10/13)	70.0% (21/30)	0.727
Live birth rate, % (n)	27.6% (8/29)	27.1% (19/70)	0.964
Obstetrics outcomes			
Babies born	8	19	​
Live birthweight (g)	3,041.3 (459.9)	3,220.0 (595.2)	0.456
Preterm birth rate, % (n)	25.0% (2/8)	15.8% (3/19)	0.616
Gestational age at delivery	38.5 (36.3.39)	38.0 (37.0.39.0)	0.729
Caesarean section rate, % (n)	87.5% (7/8)	73.7% (14/19)	0.633
Obstetric complication, % (n)	0% (0/8)	15.8% (3/19)	0.532
Birth defects	0	0	​
LBW <2,500 g, % (n)	12.5% (1/8)	15.8% (3/19)	1.000

Categorical variables were presented as n (%) and calculated by Pearson’s chi square test or Fisher’s exact test as appropriate. Continuous data was presented as mean ± SD (normally distributed) and median (25th percentile-75th percentile) (non-normally distributed). Independent samples t-test or Mann-Whitney U Test was used for the comparisons of continuous variables among groups.

HUCBMCs: Human Umbilical Cord Blood Mononuclear Cells; EMT, endometrial thickness; ES, endometrial–subendometrial; FET, frozen embryo transfer; HRT, hormone replacement therapy; GnRHa, Gonadotropin-Releasing Hormone Analogue; OI, ovulation induction; NC, natural cycle; NO., number; LBW, low birth weight.

Endometrial pattern was classified as three types: pattern A, triple line; pattern B, heterogeneous-echogen; pattern C, homogeneous-echogen.

Zone I, vessels penetrating the outer hypoechogenic area surrounding the endometrium but not entering the hyperechogenic outer margin; zone II, vessels penetrating the hyperechogenic outer margin of the endometrium but not entering the hypoechogenic inner area; and zone III, vessels entering the hypoechogenic inner area.

In terms of reproductive outcomes, no significant differences were observed between the two groups. Within the HUCBMCs group, 29 participants underwent embryo transfer (ET), resulting in 13 clinical pregnancies, 5 miscarriages, and 8 live births. Conversely, in the control group, 70 patients completed ET cycles, with 30 achieving clinical pregnancies, 9 experiencing early miscarriages, 2 experiencing late miscarriages, and 19 resulting in live births. The miscarriage rates were 38.5% for the HUCBMCs group and 36.7% for the control group (Table 3).

Notably, no obstetric complications were reported in the HUCBMCs group, whereas the control group had 2 cases of gestational hypertension and 1 case of placenta accreta (Table 3).

### The effect of HUCBMCs infusion on pregnancy outcomes

The influence of various factors on live birth is detailed in Supplementary Table S1. In the multivariate regression analysis, five variables: age, BMI, etiology of thin endometrium, endometrial pattern and the type of embryo transferred were included as adjustment factors. Blastocyst transfer was significantly associated with a higher likelihood of live birth compared to cleavage embryo transfer, as demonstrated in both crude and multivariable logistic regression analyses (OR 6.52, 95% CI 2.22–19.20; OR 7.86, 95% CI 2.20–28.08) (Supplementary Table S1).

No significant difference was observed between the HUCBMCs and control groups in terms of implantation rate (OR 0.46, 95% CI 0.16–1.32). Furthermore, the two groups showed no statistical difference in biochemical pregnancy or clinical pregnancy (OR 0.74, 95% CI 0.24–2.26; OR 0.79, 95% CI 0.26–2.41, respectively). There were also no significant differences in ongoing pregnancy or live birth between the two groups after multivariable adjustment (OR 0.96, 95% CI 0.13–7.22, OR 0.74, 95% CI 0.18–3.03, respectively) (Table 4).

**TABLE 4:** Multiple logistic regression analysis of the HUCBMCs infusion for reproductive outcomes.

​	Crude OR (95% CI)	*P*	Adjusted OR (95% CI)	*P*
Implantation	1.07 (0.49, 2.34)	0.854	0.46 (0.16, 1.32)	0.146
Biochemical pregnancy	1.02 (0.43, 2.44)	0.961	0.74 (0.24, 2.26)	0.594
Clinical pregnancy	1.08 (0.45, 2.59)	0.857	0.79 (0.26, 2.41)	0.680
Ongoing pregnancy	0.70 (0.16, 3.16)	0.643	0.96 (0.13, 7.22)	0.965
Live birth	1.02 (0.39, 2.70)	0.964	0.74 (0.18, 3.03)	0.677

The reference was the control group, the adjusted factors were maternal age, BMI, etiology of thin endometrium, endometrial pattern, the type of embryo transferred.

HUCBMCs: Human Umbilical Cord Blood Mononuclear Cells.

## Discussion

Severe thin endometrium has long been considered a major reproductive challenge for women of reproductive age, often regarded as a “terminal disease” causing infertility (Guo et al., 2019; Yu et al., 2008). The potential role of HUCBMCs as a pretreatment strategy for patients with thin endometrium undergoing FET was unclear. This study aimed to assess the impact of HUCBMCs infusion on reproductive outcomes in this patient population. Although no significant difference in live birth rate, implantation rate, or other pregnancy outcomes were observed between the HUCBMCs and the control groups, several key insights can be drawn from our findings.

HUCBMCs have been shown to promote endometrial repair through enhanced cell regeneration and reduced apoptosis. In mouse models, HUCBMCs significantly improved the proliferation and reduced the apoptosis of damaged endometrial stromal cells (ESCs), accompanied by upregulation of phospho-AKT expression while decreasing fibrosis and epithelial-mesenchymal transition-related markers (Hu et al., 2024). Additionally, the transplantation of a collagen scaffold with autologous bone marrow mononuclear cells has been shown to promote functional endometrial reconstruction by downregulating ΔNp63 expression in Asherman’s syndrome (Zhao et al., 2017). Moreover, HUCBMCs offer a more advantageous source of hematopoietic stem cells compared to adult bone marrow or peripheral blood, and HUCBMCs can be easily and conveniently isolated, thus, the study rationally proposes that HUCBMCs may represent a promising therapeutic option for treating thin endometrium.

Human umbilical cord mesenchymal stem cells (HUMSCs) have shown promising results in various preclinical and clinical trials, particularly in promoting angiogenesis and endometrial regeneration (Yang et al., 2024; Fan et al., 2024). The primary constituents of HUCBMCs are hematopoietic stem cells, mesenchymal stem cells, neural stem cells, neuron-like cells, endothelial progenitor cells, regulatory T cells, natural killer cells, T lymphocytes and dendritic cells, etc. As for the biological mechanisms which HUCBMCs are hypothesized to improve endometrial receptivity include cell- and tissue-level mechanisms. One of the primary hypothesized mechanisms is through promoting angiogenesis. Thin endometrium is characterized by poor vascular development and reduced subendometrial blood flow, which compromise oxygen and nutrient delivery to the implanting embryo (Miwa et al., 2009). Endothelial progenitor cells (EPCs) within the HUCBMC population can home to sites of ischemia and differentiate into mature endothelial cells, directly contributing to neovascularization (Duan et al., 2006). Beyond angiogenesis, immunomodulatory properties of HUCBMCs may play a critical role in optimizing the endometrial microenvironment for implantation. Thin endometrium often exhibits a chronic low-grade inflammatory state, with elevated levels of pro-inflammatory cytokines (e.g., TNF-α, IL-6) and an altered balance of uterine natural killer (uNK) cells, which are essential for spiral artery remodeling and decidualization (Robson et al., 2012). Mononuclear cells from umbilical cord blood have been shown to suppress T-cell proliferation, promote regulatory T-cell (Treg) expansion, and shift macrophage polarization from a pro-inflammatory (M1) to a pro-repair (M2) phenotype (Zhang et al., 2025). This immunomodulatory capacity could help restore endometrial immune homeostasis, creating a more permissive environment for embryo implantation. In particular, M2 macrophages are known to facilitate tissue remodeling and angiogenesis, further linking immune modulation to vascular repair. Thus, this study rationally proposes that HUCBMCs may represent a promising therapeutic option for treating thin endometrium. Nevertheless, the mechanisms of HUCBMCs are multifactorial and complex, which could explain the more modest outcomes observed in this study.

In previous studies, it was reported that human peripheral blood mononuclear cells transplantation could increase the thickness of the damaged endometrium, and exerted a positive influence on endometrial receptivity and embryonic implantation in mice. Lingjuan Wang’s study on rhesus monkeys with intrauterine adhesion (IUA) demonstrated that HUCBMCs, hyaluronic acid gel (HA-GEL), and the huMSC/HA-GEL complexes could partially repair IUA caused by mechanical injury (Wang et al., 2020). While our study confirmed the safety of intrauterine HUCBMCs infusion, no significant improvements in live birth rate and other reproductive outcomes were revealed. First, a subset of cells may have lost potency during cryopreservation or failed to adhere to the endometrial surface. Some studies have applied collagen scaffolds and autologous bone marrow mononuclear cells to stimulate functional endometrial hyperplasia. If the adhesion of HUCBMCs to the uterine cavity or their retention time can be increased, it may yield better effects in stimulating functional endometrial reconstruction (Zhao et al., 2017). In addition, some studies have applied a urinary catheter balloon to block the cervix before intrauterine infusion to prolong the time that stem cells stay in the uterine cavity (Huang et al., 2022).

Second, the timing of HUCBMCs administration may not have aligned with the optimal endometrial repair window. Our protocol involved HUCBMCs infusion immediately after endometrial scratching on menstrual cycle days 3–4, a period corresponding to the early follicular phase when the endometrium is in a state of shedding and initial regeneration. However, there is literature indicating that stem cell perfusion was performed on the 10th day of the menstrual period (Ding et al., 2014). In some animal studies, perfusion was carried out during the estrous cycle (Ding et al., 2014). Additionally, stem cells are expensive and difficult to obtain, and whether multiple perfusions are more beneficial for endometrial hyperplasia has not been verified in this study. Therefore, more research is needed to confirm the timing and frequency of perfusion.

Patient heterogeneity could also have masked potential therapeutic effects or contributed to variable responses. Patients with severe fibrosis may have impaired tissue plasticity, preventing HUCBMCs from inhibiting epithelial-mesenchymal transition (EMT) or promoting stromal cell regeneration as observed in previous studies (Hu et al., 2024; Zhao et al., 2017). Finally, the mechanistic pathways underlying HUCBMCs action may not have been sufficiently activated in our patient cohort. Since all ETs were performed within the same treatment cycle, which precluded the collection of endometrial tissue for histological or molecular analysis. Consequently, our interpretation of the endometrial receptivity markers is necessarily restricted. In summary, the lack of significant reproductive benefits with HUCBMCs infusion in our study is likely multifactorial, involving suboptimal cell dose/composition, misalignment with the endometrial repair window, unaccounted patient heterogeneity, and incomplete activation of mechanistic pathways. Future studies should address these limitations by optimizing cell dose, refining administration timing, stratifying patients by fibrosis severity, and integrating molecular assays to validate mechanistic engagement, all of which may help unlock the therapeutic potential of HUCBMCs for thin endometrium.

Several alternative treatments, such as platelet-rich plasma (PRP) and sildenafil citrate (Yang et al., 2025) (Moini et al., 2020), have also been shown to improve endometrial receptivity in women with thin endometrium. These treatments work by enhancing endometrial blood flow and stimulating tissue regeneration, mechanisms that may overlap with those of HUCBMCs. However, HUCBMCs contain a broader range of bioactive components, including immune cells and endothelial progenitors, which might offer additional benefits in tissue repair and immune modulation, even if their direct impact on endometrial is less pronounced.

Furthermore, our study found that miscarriage rates of patients with thin endometrium were more than 36% in both groups, which is much higher than tubal infertility couples in China (3.4%) (Zheng et al., 2019). As far as we know, EMT is strongly associated with pregnancy losses and live births in IVF. But the reason for why a thin endometrium leads to implant failure or early pregnancy loss still remains unclear. A thin or absent functional layer may subject the embryos to higher vascularity and oxygen concentrations from the basal endometrium. As a result, the subsequent production of reactive oxygen species could generate a suboptimal uterin environment for implantation and placentation, which ultimately leads to impaired fetal growth (Zhang et al., 2019). Xu, J et al. showed that EMT exhibits a curvilinear relationship with pregnancy outcomes in fresh embryo transfer cycles. When EMT ≥ 12 mm, clinical pregnancy rate, live birth rate and miscarriage rate may achieve their optimal level (Xu et al., 2021). The miscarriage rate in previous studies was 15.6%–26.7% (Xu et al., 2021; Yuan et al., 2016; Ma et al., 2017; Craciunas et al., 2019), which is much lower than our study. This observed outcome may be related to our participants’ thinner EMT. Further studies are necessary to explore the mechanism of thin endometrium on the miscarriage rate following FET.

Our study also showed that blastocyst transfer was significantly associated with a higher live birth rate compared to cleavage-stage embryo transfer. This finding is consistent with existing literature, which suggests that blastocyst-stage embryos are more likely to implant and result in successful pregnancies, owing to their advanced developmental stage, lower aneuploid rate and improved synchronization with the endometrial window of implantation (Liu et al., 2025). Besides, an increasing number of studies have placed emphasis on cumulative live-birth rates. It proved single blastocyst transfer increases cumulative live-birth rates over single cleavage-stage transfer (Ma et al., 2024). For women with thin endometrium who undergo frozen embryo transfer, blastocyst transfer may be a better option.

Of course, there are also some limitations to this study. Firstly, this study was limited by a small sample size, resulting in insufficient statistical power. As demonstrated by our *post hoc* power analysis, this study’s power was extremely low (0.051). Smaller, yet clinically relevant, improvements would likely have gone undetected, leading to a high risk of Type II error. Therefore, the absence of statistically significant differences should not be interpreted as definitive evidence of no treatment effect. Rather, these findings should be considered preliminary and hypothesis-generating, underscoring the need for larger, adequately powered randomized controlled trials to definitively evaluate the efficacy of HUCBMCs infusion. The lack of randomization can introduce a potential for selection bias. Secondly, the dosage of HUCBMCs, the method of administration, and the optimal timing warrant more research. Thirdly, the retained time of HUCBMCs in the uterine was no more than 24 h, which would affect the therapeutic potential. Hence, clinical-grade HUCBMCs loaded onto a 3D printed scaffolds according to the volume and shape of uterine and cesarean scar diverticulum based on medical imaging will prolong the retained time. Finally, since all ETs were performed within the same treatment cycle, which precluded the collection of endometrial tissue for histological or molecular analysis. Consequently, ultrasound scanning was used to detect endometrium receptivity but molecular marker.

Despite the absence of significant difference between the HUCBMCs and control group, our study offers valuable insights into the clinical application of HUCBMCs for patients with thin endometrium. Future research involving larger cohorts and longer follow-up periods is warranted to evaluate potential long-term benefit and to optimize treatment strategies for this promising cell-based therapy.

## Conclusion

In summary, while HUCBMCs did not significantly improve pregnancy outcomes in patients with thin endometrium undergoing FET, they represent a novel and safe therapeutic approach worthy of further investigation. Additional studies are necessary to fully elucidate their potential and to compare their efficacy with other regenerative therapies, such as HUCBMCS, PRP, and growth hormone.

## Data Availability

The raw data supporting the conclusions of this article will be made available by the authors, without undue reservation.
