# Supplementary material for: Impact of intrauterine infusion of human umbilical cord blood mononuclear cells on reproductive outcomes in patients with thin endometrium undergoing frozen embryo transfer
Source: Front Cell Dev Biol. 2026 May 28;14:1758224. doi: 10.3389/fcell.2026.1758224 (PMC13253671; doi:10.3389/fcell.2026.1758224)
Supplement: Supplementary file 1 [file Table1.docx]

**Supplementary Table S1** The effect of variables on live birth in univariate and multivariate logistic regression analysis in the study.

| Variables | Univariate | *P* | Multivariate | *P* |
| --- | --- | --- | --- | --- |
|  | Crude OR(95%CI) |  | Adjusted OR(95%CI) |  |
| Group | 1.02 (0.39,2.70) | 0.964 | - | - |
| Age | 0.81 (0.71,0.93) | 0.002 | 0.87 (0.75,1.00) | 0.058 |
| BMI(kg/㎡) | 0.77 (0.65,0.92) | 0.003 | 0.86(0.71,1.03) | 0.103 |
| Infertility type |  |  |  |  |
| Primary | Reference |  |  |  |
| Secondary | 0.57 (0.21,1.58) | 0.281 | - |  |
| Infertility duration (years) | 1.05 (0.90,1.23) | 0.516 | - |  |
| No. of previous curettages | 0.49 (0.31,0.78) | 0.003 | - |  |
| Etiology |  |  |  |  |
| Traumatic | Reference |  |  |  |
| Other cause | 3.00 (1.20,7.53) | 0.019 | 2.79 (0.84, 9.31) | 0.095 |
| Failed ET cycles | 0.85 (0.55,1.30) | 0.446 | - |  |
| EMT(mm) | 1.35 (0.76,2.39) | 0.305 | - |  |
| Patients with good quality embryos | 1.29 (0.52,3.19) | 0.589 | - |  |
| Endometrial pattern |  |  |  |  |
| Pattern A | Reference |  |  |  |
| Pattern B | 0.27 (0.10, 0.75) | 0.012 | 0.26 (0.07,0.90) | 0.033 |
| Pattern C | 0 | 0.989 | 0 | 0.992 |
| ES blood flow distribution pattern |  |  |  |  |
| Zone I | Reference |  |  |  |
| Zone II | 3.54 (0.73, 17.27) | 0.118 | - |  |
| Zone III | 4.47 (0.86, 23.37) | 0.076 | - |  |
| No. of embryos transferred | 0.40 (0.16,1.03) | 0.057 | - |  |
| Stage of embryos transferred |  |  |  |  |
| Cleavage day-3, n(%) | Reference |  | Reference |  |
| Blastocyst, n(%) | 6.52 (2.22,19.20) | ＜0.001 | 7.86 (2.20, 28.08) | 0.002 |
| FET Protocols |  |  |  |  |
| HRT | Reference |  |  |  |
| GnRHa+HRT | 0 | 0.999 | - |  |
| OI | 0.83 (0.20,3.42) | 0.798 | - |  |
| NC | 0.37 (0.42,3.25) | 0.369 | - |  |

In the multivariate model, the adjusted factors were maternal age, BMI, etiology of thin endometrium, endometrial pattern, the type of embryo transferred.

HUCBMCs: Human Umbilical Cord Blood Mononuclear Cells; EMT, endometrial thickness; ES, endometrial–subendometrial; FET, frozen embryo transfer; HRT, hormone replacement therapy; GnRHa, Gonadotropin-Releasing Hormone Analogue; OI, ovulation induction; NC, natural cycle; NO., number.

**Supplementary Table S2** Serum levels of WBC count, neutrophil percentage and C-reactive protein at oneweek after HUCBMCs intrauterine infusion in each patient.

|  | WBC count  (10^9/L) | Neutrophil percentage  (%) | C-reactive protein (mg/L) |
| --- | --- | --- | --- |
| P1 | 5.8 | 55.9 | 1.7 |
| P2 | 6.4 | 64.7 | 1.6 |
| P3 | 6.3 | 67.2 | 2.5 |
| P4 | 4.9 | 53.7 | 2.3 |
| P5 | 6.9 | 62.2 | 2.7 |
| P6 | 6.0 | 70.3 | 3.1 |
| P7 | 3.7 | 54.1 | 2.2 |
| P8 | 5.1 | 59.2 | 2.9 |
| P9 | 6.3 | 60.9 | 2.2 |
| P10 | 4.8 | 50.0 | 2.7 |
| P11 | 6.5 | 57.4 | 2.8 |
| P12 | 5.8 | 55.9 | 2.1 |
| P13 | 5.6 | 53.3 | 2.5 |
| P14 | 8.3 | 63.1 | 2.2 |
| P15 | 9.6 | 74.6 | 3.3 |
| P16 | 5.3 | 65.4 | 2.6 |
| P17 | 6.6 | 62.3 | 1.9 |
| P18 | 7.8 | 66.6 | 2.4 |
| P19 | 7.4 | 52.9 | 3.2 |
| P20 | 5.1 | 56.8 | 2.8 |
| P21 | 8.9 | 64.4 | 2.6 |
| P22 | 6.8 | 68.8 | 2.3 |
| P23 | 8.1 | 67.2 | 2.2 |
| P24 | 6.9 | 62.6 | 1.9 |
| P25 | 8.7 | 66.8 | 2.0 |
| P26 | 7.9 | 58.8 | 2.6 |
| P27 | 7.2 | 63.5 | 1.8 |
| P28 | 5.6 | 62.1 | 2.7 |
| P29 | 7.4 | 66.5 | 2.3 |
| P30 | 6.8 | 63.4 | 1.8 |
| P31 | 7.3 | 66.7 | 1.6 |
| P32 | 7.8 | 56.8 | 2.1 |
| P33 | 5.6 | 78.7 | 2.4 |
| P34 | 6.4 | 67.5 | 2.2 |
| P35 | 4.9 | 59.6 | 1.9 |

WBC, white blood cell.
